# Transcriptomes of *Trypanosoma brucei rhodesiense* from sleeping sickness patients, rodents and culture: Effects of strain, growth conditions and RNA preparation methods

**DOI:** 10.1371/journal.pntd.0006280

**Published:** 2018-02-23

**Authors:** Julius Mulindwa, Kevin Leiss, David Ibberson, Kevin Kamanyi Marucha, Claudia Helbig, Larissa Melo do Nascimento, Eleanor Silvester, Keith Matthews, Enock Matovu, John Enyaru, Christine Clayton

**Affiliations:** 1 Centre for Molecular Biology of Heidelberg University (ZMBH), DKFZ-ZMBH Alliance, Im Neuenheimer Feld 282, Heidelberg, Germany; 2 Department of Biochemistry and Sports Science, College of Natural Sciences, Makerere University, Kampala, Uganda; 3 Bioquant, Im Neuenheimer Feld 267, Heidelberg, Germany; 4 Institute for Immunology and Infection Research, School of Biological Sciences, University of Edinburgh, Edinburgh, United Kingdom; 5 Department of Biotechnology and Diagnostic Sciences, College of Veterinary medicine, Animal resources and Biosecurity, Makerere University, Kampala, Uganda; McGill University, CANADA

## Abstract

All of our current knowledge of African trypanosome metabolism is based on results from trypanosomes grown in culture or in rodents. Drugs against sleeping sickness must however treat trypanosomes in humans. We here compare the transcriptomes of *Trypanosoma brucei rhodesiense* from the blood and cerebrospinal fluid of human patients with those of trypanosomes from culture and rodents. The data were aligned and analysed using new user-friendly applications designed for Kinetoplastid RNA-Seq data. The transcriptomes of trypanosomes from human blood and cerebrospinal fluid did not predict major metabolic differences that might affect drug susceptibility. Usefully, there were relatively few differences between the transcriptomes of trypanosomes from patients and those of similar trypanosomes grown in rats. Transcriptomes of monomorphic laboratory-adapted parasites grown in *in vitro* culture closely resembled those of the human parasites, but some differences were seen. In poly(A)-selected mRNA transcriptomes, mRNAs encoding some protein kinases and RNA-binding proteins were under-represented relative to mRNA that had not been poly(A) selected; further investigation revealed that the selection tends to result in loss of longer mRNAs.

## Author summary

African trypanosomes cause sleeping sickness in humans and various diseases of domestic and wild animals. Until now, all of our current knowledge of African trypanosome metabolism is based on results from trypanosomes grown in *in vitro* culture or in rodents. Drugs against sleeping sickness must however treat trypanosomes in humans. We here examine the way in which genes are expressed in human sleeping sickness trypanosomes from the blood and cerebrospinal fluid of human patients, and compare our results with those from trypanosomes growing in culture and rodents. Gene expression profiles of trypanosomes from human blood and cerebrospinal fluid were quite similar and there was no evidence for differences that might affect drug susceptibility. The RNAs in laboratory-adapted parasites grown in *in vitro* culture also quite closely resembled those in parasites from humans. The results showed that technical differences in the way RNA is made can have strong effects on measured gene expression profiles.

## Introduction

*Trypanosoma brucei* subspecies and related parasites infect humans, cattle, camels, and horses, causing substantial economic losses throughout the tropics [1, 2]. Human sleeping sickness in East Africa is caused by *Trypanosoma brucei rhodesiense*, a zoonotic parasite which differs from *Trypanosoma brucei brucei* (which is found in cattle) only by the acquisition of a single gene enabling survival in human serum [3]. *Trypanosoma brucei gambiense* causes a more chronic human disease in West Africa. After an initial phase in which the parasites are restricted to the blood and tissue fluids, *T*. *gambiense* and *T*. *rhodesiense* penetrate the central nervous system (CNS). *T*. *rhodesiense* disease is usually fatal, whereas some *T*. *gambiense*-infected people are asymptomatic [4]. The organisms completely evade adaptive humoral immunity because they show antigenic variation, repeatedly changing their surface coat of variant surface glycoprotein (VSG). As a consequence, disease control has to rely on chemotherapy of detected cases, combined with insecticides and traps to control the tsetse fly vector. There are, however, very few drugs available to treat African trypanosomiasis, they are all unacceptably toxic, and resistance is arising [5]. Moreover, within the CNS, trypanosomes are sensitive only to drugs that cross the blood-brain barrier, limiting therapeutic options for the late stage of the disease.

All of our knowledge of the biochemistry and molecular biology of *T*. *brucei* depends on laboratory models, and this includes the early phases of drug development. Targeted approaches rely on biochemical knowledge gained from culture alone for target selection; in the phenotypic approach, compounds are initially screened using cultured trypanosomes. Promising leads are then tested in rodent models. Within rodents, as in other mammals, *T*. *brucei* spread throughout the blood and tissue fluids and invade the brain. Most trypanosomes within the rat brain parenchyma appear degraded, although cells of normal appearance are seen in the pia mater and cerebrospinal fluid (CSF) [6]. Many trypanosomes are also found in the adipose tissue of mice; in this case, transcriptome analysis suggested metabolic differences from blood trypanosomes [7]. It is therefore possible that differences between trypanosomes at different sites could contribute to treatment failure.

In natural *T*. *brucei* infections, the trypanosomes are pleomorphic. Proliferating forms have long slender morphology, and obtain ATP through glycolysis. The parasites produce a soluble signal known as "Stumpy Inducing Factor" (SIF), whose identity is still unknown [8, 9]. At high density, when the SIF concentration reaches a critical threshold, the trypanosomes arrest in G1, and acquire a more stumpy shape [10–12]. Stumpy forms have increased expression of some mitochondrial proteins; markers that are absent (or much less expressed) in bloodstream forms include ESAG9 [13], PAD1 [14] and the protein phosphatase PIP39 [15]. The higher expression of mitochondrial protein mRNAs means that stumpy forms are pre-adapted to differentiate into the procyclic form which multiplies in the tsetse midgut, since procyclic forms rely on mitochondrial energy metabolism. Procyclic forms lack VSG, instead having a surface coat of procyclin proteins containing Glu-Pro (EP) or Gly-Pro-Glu-Glu-Thr (GPEET) repeats. Relative to long slender forms, stumpy forms have slightly increased procyclin mRNA expression [16, 17], although the protein is not made.

The density at which long slender trypanosomes cease to proliferate, and become stumpy, differs according to the environment [8]. In rodents, maximal densities of fully pleomorphic parasites are 1–5 x 10^8^/ml [16–18] for the initial parasitaemia, with differentiation initiating above 5 x 10^7^/ml. In contrast, differentiation-competent trypanosomes in liquid culture arrest at 1–2 x 10^6^/ml [8]). In experimentally infected cattle [19] or *Mastomys natalensis* rats (but not Swiss mice) [18], parasitaemias are lower during chronic infection than in the initial wave. Humans who present for sleeping sickness diagnosis, have usually been infected for some time, and rarely show *T*. *rhodesiense* parasitaemias above 10^6^/ml, although a parasitaemia of 10^8^/ml was recently recorded in a Polish tourist [20]. The reasons for the low densities during chronic infection are unknown. The infection may be suppressed via innate immunity and inflammatory responses; there may be metabolic constraints; or SIF may accumulate more readily. In cattle, the infectivity of the parasites for tsetse seems to be relatively unaffected by the parasitaemia level [19]. Perhaps SIF levels in cattle are high despite low parasitaemias, or the trypanosomes are more receptive to it than in the initial wave; or alternatively there may be tissues in which parasite densities are substantially higher than in the blood.

After multiple passages in rodents or culture, African trypanosomes lose the ability to make stumpy forms, becoming monomorphic. It is these forms that are used for most biochemical and molecular biology experiments.

In the work described in this paper, we set out to characterize *T*. *rhodesiense* in human patients. We asked two questions:

a) Do trypanosomes in the CSF differ from those in the blood?
b) Do trypanosomes in humans differ from trypanosomes in culture or in rodent blood?

To answer these questions, one should ideally compare the proteomes of different parasites growing in different environments. Messenger RNA levels do not predict protein levels reliably, because trypanosomes have strong regulation of translation [21–23], and protein degradation rates are presumably also important. However, due to the very limited amount of material available, and the small numbers of parasites in comparison with host cells, proteome characterization of trypanosomes from patients is not feasible. We therefore instead analyzed transcriptomes. The results suggest that cultured trypanosomes are in most respects a satisfactory model for parasites in humans. The gene expression profiles also indicated that parasites in human CSF are, if anything, growing more actively than those in human blood.

## Results and discussion

### Sleeping sickness transcriptome collection

Samples of blood and CSF were obtained from patients presenting for diagnosis at the clinic in Lwala hospital, Kaberamaido district, which is located in the *T*. *b*. *rhodesiense* focus of North Eastern Uganda. We tested a variety of methods for RNA preparation using mixtures of trypanosomes with blood from the Heidelberg University blood bank. All gave acceptable yields of intact RNA, with the best results being obtained by suspension of buffy coat trypanosomes in denaturing solutions such as Trizol. In contrast, use of such methods with field blood samples resulted in exceptionally low yields of RNA (a few nanograms), and the preparations were much too degraded to allow RNASeq library preparation. This was true even if the purified RNA was initially resuspended in a solution containing RNase inhibitors.

We succeeded in obtaining sufficient RNA for sequencing from blood only when the samples were placed directly into PAXgene tubes [24]. For CSF, some RNAs were purified using Trizol and some using the PAXgene tubes. Samples from blood with the highest trypanosome densities, and from CSF with highest ratios of trypanosomes to leukocytes, were chosen for RNA preparation. For CSF, human rRNA was depleted; for the blood mRNA samples, both rRNA and haemoglobin mRNA were depleted. Subsequently cDNA libraries were prepared and sequenced. To simplify the alignment and counting, an easy-to use pipeline was created; this can be downloaded from [25]. Within this pipeline, the sequences were first trimmed to exclude the 60 most abundant sequences; these include not only the adapters, but also the most over-represented rRNAs. Removal of these over-represented sequences greatly simplified and sped up the subsequent alignment. After alignment and read counting, libraries from samples giving adequate numbers of trypanosome reads were re-sequenced to increase the read depth. All of the resulting datasets are available at Array Express.

For CSF, parasitaemias varied from 4–66 x 10^4^/ml, and between 1% and 10% of reads were trypanosome-specific. These numbers roughly correlated with the ratio of trypanosomes to white blood cells, and suggested that a CSF white blood cell contained about 10 times more mRNA than the trypanosomes (Table 1 and S1 Table, sheet 1). PAXgene sampling lyses the parasites. This meant that all blood parasitaemias had to be estimated using the diagnostic thin films, using rat samples for calibration. Bloodstream parasitaemias were 100–1000 times higher than those in CSF, and the percentage of reads aligning to the *T*. *brucei* TREU927 genome varied between 6% and 77% (Table 1 and S1 Table, sheet 1), but these reads included a substantial (and variable) proportion that corresponded to rRNA. For comparison, when poly(A)+ RNA from *Leishmania brazilienesis* mouse skin lesions was sequenced, about 1% of the reads mapped to the *Leishmania* genome [26]. In this paper, we discuss the trypanosome transcriptomes. Results for the human mRNAs will be analysed separately.

**Table 1:** Human samples. Samples labelled "HC" were from CSF, and those labelled "HB" were from blood. WBC = white blood cells, Tryp = trypanosome, and numbers are cells/ml x 10–4. na = not available. For blood, counts were estimated from stained thin smears. More details are in S1 Table.

No.	WBC /ml (x10^-4^)	Tryp /ml (x10^-4^)	RNA (μg)	Reads (x10^-7^)	Tryp reads (x10^-6^)	% reads tryp	Tryps/ WBC
HC50	12	4	0.4	19.94	8.46	4%	0.35
HC57	38	13	0.2	18.12	11.74	6%	0.33
HC58	84	66	0.2	24.37	28.53	12%	0.78
HC60	120	8	0.7	3.29	0.43	1%	0.07
HC69	na	4	0.1	3.64	0.31	1%	na
HC71	na	50	0.5	18.51	20.35	11%	na
HB69	na	327	7.6	3.64	0.06	0.2%	na
HB72	na	1600	8	15.30	0.67	0.4%	na
HB74	na	1300	8.6	12.30	94.71	77%	na
HB73	na	4500	8.1	10.29	19.12	19%	na
HB71	na	5100	8	14.21	8.56	6%	na
HB80	na	2100	2.6	7.65	25.56	33%	na
HB81	na	1100	3.0	8.95	32.90	36%	na

### Trypanosomes from infected rats

**Fig 1:**
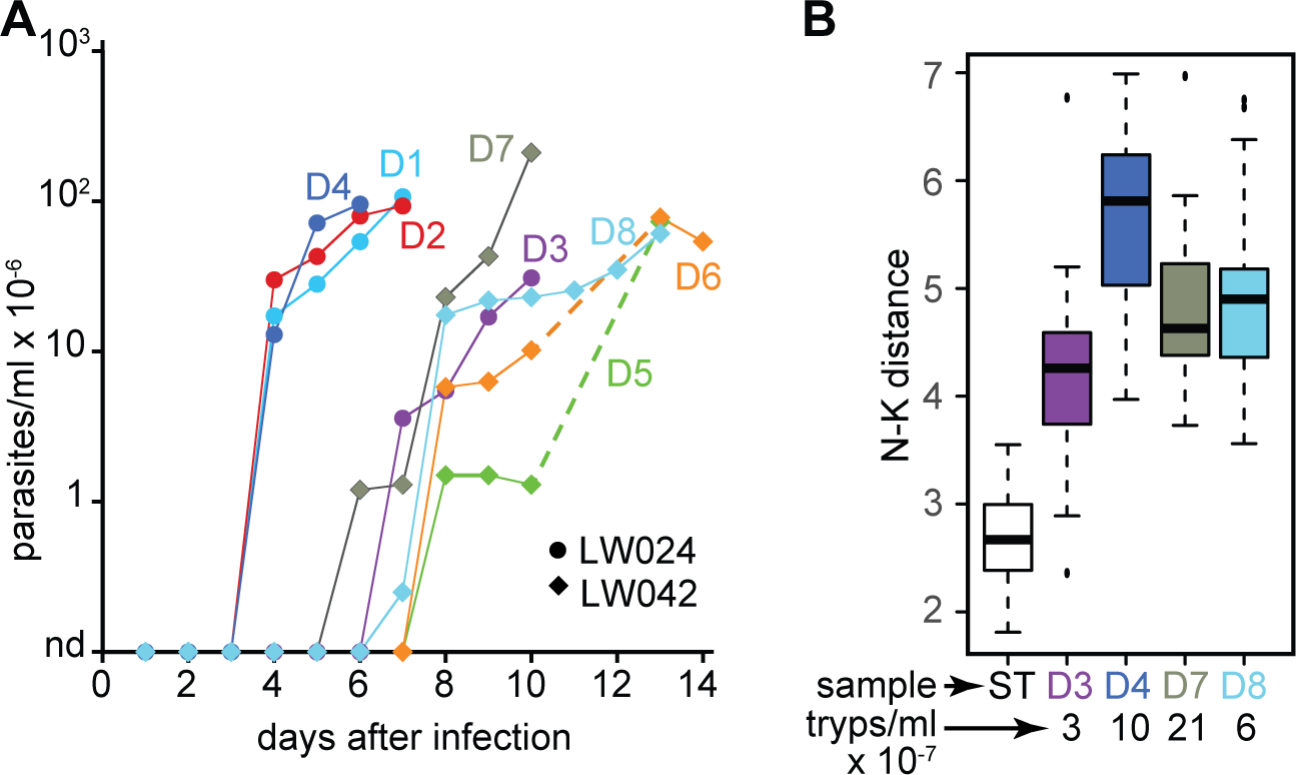
Rat infections. A. Parasitaemias of infected rats. B. Morphological analysis: distance between the kinetoplast and the nucleus. Between 24 and 59 parasites were counted on thin blood films stained for DNA; results for 8 PAD1-positive EATRO 1125 stumpy forms (ST, made in stationary phase cultures) were included for comparison and as a positive control for PAD1 staining. Parasite outlines were detected by a combination of tubulin staining, PAD1 background, and differential interference microscopy.

To enable direct comparison of the human results with an experimental sample obtained using exactly the same methods, we infected 8 immunocompetent rats with two *T*. *rhodesiense* isolates that we had obtained 1–2 years earlier from patients attending the same clinic as the current ones [17]. These trypanosomes had undergone 2 mouse passages prior to infection; their genomes are similar to each other [17]. Transcriptomes from single rats had been obtained from these lines previously (RBC1 and RBC4 in S1 Table), but those parasites were morphologically uncharacterized and parasitaemias were unknown [17]. This time, blood for RNA preparation was taken at parasitaemias ranging from 5 x 10^7^–2 x10^8^ (Fig 1A and S1 Table sheet 1; samples "RBD"). RNA was prepared from all samples and treated exactly as for human blood. Sufficient reads for analysis were obtained from six samples.

After infection of immunocompetent mice with EATRO1125 cells, *PAD1* mRNA became detectable when parasitaemias attained about 2 x 10^8^/ml, and stumpy forms were present when this density had persisted for 3 days [27]. To see whether the newly-isolated trypanosomes behaved similarly in rats, thin blood films from four rats were stained for PAD1 and for DNA, and the distance between the nucleus and kinetoplast was measured. We did not detect any stumpy forms: the cells were longer than stumpy forms and no PAD1 was detected (Fig 1B). We also searched three of the relevant raw sequence files for short sequences specific to *PAD1* [27], but could not find any matches. This suggests that in trypanosomes from this region, the *PAD* gene family has diverged too far to allow *PAD1* identification from sequence alone. Unexpectedly, there was no correlation between cell density and parasite length (Fig 1B). In the immunocompetent rats the immune response was presumably contributing to parasitaemia control.

Sample RBD3, which had the shortest parasites among the three tested samples, had relatively high levels of *PAD* gene family mRNA (S1 Table sheet 2), although we were unable to detect PAD1 protein or *PAD1*-specific sequence. This sample unfortunately yielded too few trypanosome-derived reads for statistically valid transcriptome analysis. The low read count was presumably partly caused by the low parasitaemia, but since it is known that stumpy forms have low mRNA content [28], this might also have contributed.

### Trypanosomes from other sources

In addition to the samples described above, we incorporated three previously published datasets for blood trypanosomes from rats infected with either culture-adapted or fresh *T*. *rhodesiense*, and several datasets from mouse blood. Three of the mouse transcriptomes were new data for well-characterised long slender trypanosomes of a pleomorphic strain (samples "MBA"). Data for mouse adipose tissue (Mad) were also included. Finally, a variety of published results from cultured parasites (Cult) were included; these were almost uniformly from cells at low enough densities to be in log phase. Details of all datasets studied are in Table 2 and further information is in S1 Table, sheet 2. Raw sequence data that had been obtained from other labs were re-analysed using our own pipeline in order to ensure that parameters for alignment and read counting were identical.

**Table 2:** Compared datasets. Laboratories are: 1: Mulindwa, Makerere University; 2: Clayton, ZMBH; 3: Figueiredo, University of Lisbon; 4: Matthews, Edinburgh University. Details of individual samples are in S1 Table.

CODE	Lab	Strain	type	source	Cell density	purification	reference
CultA	2	Tb Lister 427	monomorphic	culture	1–1.5 x 10^6^	Ribo-minus eukaryote kit (Invitrogen)	[40]
CultB	2	Tb Lister 427	monomorphic	culture	1 x 10^6^	poly(A)+	[40]
CultC	2	Tb Lister 427	monomorphic, 5 min sinefungin	culture, 5 min Sinefungin	1–1.5 x 10^6^	Ribo-minus eukaryote kit (Invitrogen)	[35]
CultD	2	Tb Lister 427	monomorphic	culture	1–2 x 10^6^	ribo- & poly(A)+	[32]
CultE	2	Tb Lister 427	monomorphic	culture	1–1.5 x 10^6^	poly(A)+	[60]
CultF	2	Tb Lister 427	monomorphic	culture	1–1.5 x 10^6^	Ribo-minus eukaryote kit (Invitrogen)	[61]
CultG	2	Tb EATRO 1125	lab-adapted pleomorphic	culture	7 x 10^5^	poly(A)+	[62]
RBA	1	Tbr729	culture-adapted	Rat buffy coat	2–5 x 10^8^	poly(A)+	[63]
RBB	1	Tbr729	culture-adapted	Rat buffy coat DEAE	2–5 x 10^8^	poly(A)+	[63]
RBC	1	4 new Tbr	2 mouse passages	Rat buffy coat	2–5 x 10^8^	poly(A)+	[17]
RBD 1–4	1	Tbr patient 24	2 mouse passages	Rat blood	0.3–1 x 10^8^	Ribo-globin-clear	This paper
RBD 5–8	1	Tbr patient 42	2 mouse passages	Rat blood	0.5–5 x 10^8^	Ribo-globin-clear	This paper
MBA	4	Tb EATRO 1125	Long slender, lab-adapted pleomorphic	Mouse blood DEAE	2–7 x 10^7^	poly(A)+	This paper
MBB	3	Tb EATRO 1125	lab-adapted pleomorphic	Mouse blood DEAE	4–17 x 10^7^	ribo- & poly(A)+	[7]
MAd	3	Tb EATRO 1125	lab-adapted pleomorphic	Mouse adipose tissue	10^4^−10^5^	ribo- & poly(A)+	[7]

Most of the transcriptomes had been prepared using mRNA that had been purified either by poly(A) selection (giving "poly(A)+" RNA), or by depletion of rRNA (giving "ribo-minus" RNA). Both selections involve hybridization to oligonucleotides coupled to magnetic beads. For poly(A) selection the RNA is bound to oligo d(T) in high salt, washed with lower salt buffer, then eluted with water. For rRNA depletion, a set of oligonucleotides complementary to rRNA is used, with moderate salt conditions; the RNA is allowed to bind, then the supernatant is taken for further analysis.

### Applications for data analysis

To align the transcriptomes, we wrote various user-friendly scripts. First, there is a python script that aligns the reads while allowing for the peculiar nature of kinetoplastid genomes [25]. Since mRNA annotation is incomplete and many mRNAs have numerous different processing sites, the script counts only the reads that align to open reading frames. Trypanosome genomes have many repeated genes, so to account for this, the application is set to allow each read to align up to 20 times. In the subsequent analysis, over-counting of the repeated genes is avoided by considering only a set of "unique" open reading frames in which only one representative of each sequence was present. (This list is adapted from [29]). As a consequence, the RNA abundances for each gene, as estimated by reads per million reads (RPM), if normalized to open reading frame length, should approximate to the level of mRNA.

To analyse the data statistically we used another custom application, DEseqU1, which runs in RStudio and uses DEseq2 [30] for significance estimation. The application yields principal component analysis, which shows which transcriptomes are closely related. In addition, it allows analysis according to gene functions and cell cycle regulation [31]. We assigned gene functions using a combination of the annotations in TritrypDB, and manual annotations based on publications; all are listed in S1 Table, sheet 3. Both the unique gene list and the assigned gene functions can be changed by editing the relevant text files.

Finally, heat maps were generated using another RStudio script, ClusterViewer.rmd. This is included as S1 app. The included folder enables readers to examine the data in this paper for themselves, either by looking at all genes, or by examining specific functional categories. ClusterViewer can be adapted easily for other datasets by changing the input file and a few lines of the script, as described in the instructions.

The unique gene list does not include variant surface glycoproteins (VSGs). To find VSGs expressed in human patients, we took the two largest datasets and assembled all mRNAs as contigs. Next, we searched for the 13nt sequence that is shared by the 3'-untranslated regions. The pipeline used to do this is at https://github.com/klprint/IdentifyVSGs and the assembled VSGs are in the supplement.

### Preliminary comparisons: mRNA selection and strain

All read counts are presented in S1 Table, sheet 3. Normalized values (reads per million reads) are in S1 Table, sheet 4. Only datasets with at least 3x10^5^ reads aligning to the unique gene set were analysed (S1 Table, sheet 1); this resulted in exclusion of two samples for each set of field isolates.

**Fig 2:**
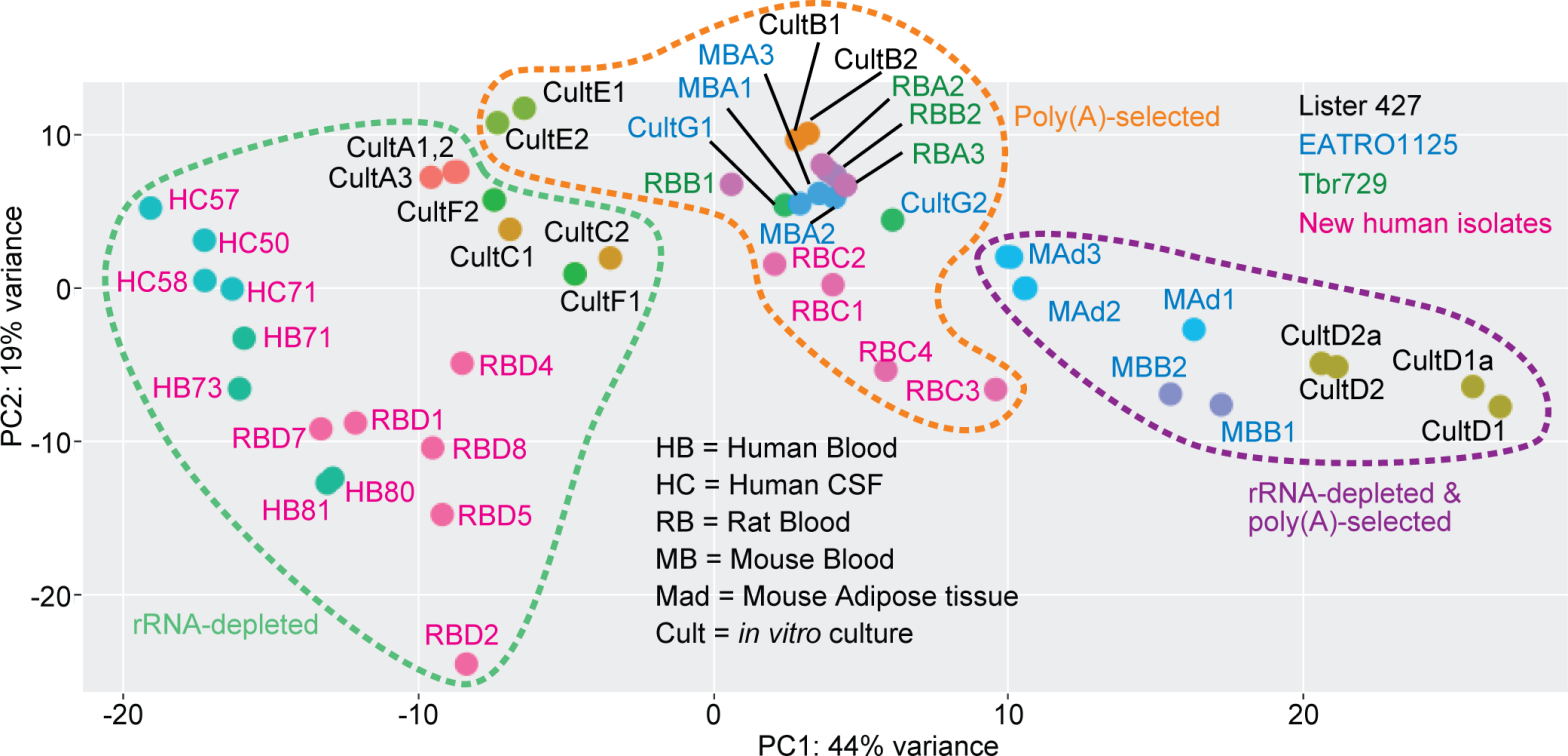
Relationships between transcriptomes of *T*. *brucei* and *T*. *rhodesiense* from different growth environments. Principal component analysis is shown for all analysed datasets. Samples are colour-coded according to origin, and individual samples are labelled on the plot. The dotted lines indicate three different methods to enrich mRNA prior to sequencing. Details of the samples are in S1 Table, sheet 1 and in Tables 1 and 2.

The principal component analysis in Fig 2 shows how the different transcriptomes are related. It covers 63% of the total variance, with 44% of the variation on the x-axis. Strikingly, mRNAs prepared in similar ways mostly clustered together irrespective of source (Fig 2). The only exception was one poly(A)+ culture dataset (CultE), which was quite similar to the ribo-minus culture transcriptomes; the reason for this is unknown and it will not be considered further.

Among the poly(A)+ mRNAs, transcriptomes from four rats infected with recent Ugandan isolates [17] showed considerable variation; RBC1 and RBC2 were extremely similar to those of from a culture-adapted Ugandan strain (RBA) and from long slender EATRO1125 strain trypanosomes in mice (MBA), whereas two samples that had relatively high expression of stumpy-form marker mRNAs (RBC3 and RBC4) [17] were somewhat apart from the main cluster.

Transcriptomes that had been generated using RNA that was both poly(A) selected and rRNA depleted clustered separately from the others. As reported previously, within this set there were differences between cells from adipose tissue, blood and culture [7]. However, the results from cultured cells [32] were surprisingly different from others despite similar growth conditions (S1 Table Sheet 2). This discrepancy is presumably due to technical differences, so these datasets were not included in subsequent comparisons.

### Are longer mRNAs selectively lost during poly(A) selection?

**Fig 3:**
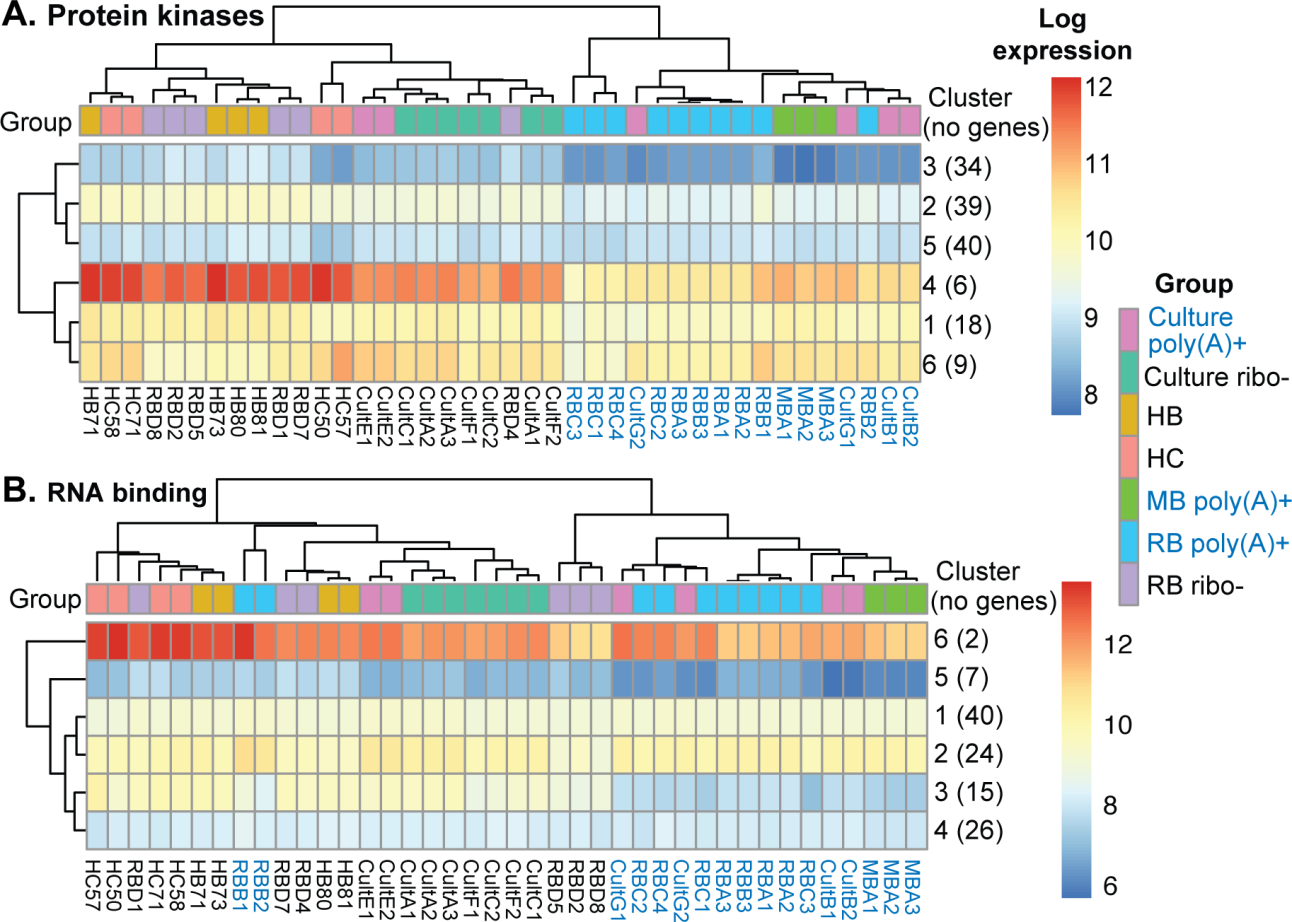
Some mRNAs encoding regulatory proteins are lost during poly(A) selection. The heat maps show relative expression (log_2_ values from DeSeq2) for mRNAs encoding protein kinases (A) and RNA binding proteins (B). Poly(A)+ samples are labelled in blue. Detailed results for protein kinase cluster 4 and RNA-binding protein cluster 3 are in S2 Table, sheet 3.

Before comparing the human samples with the others, we looked at the effects of poly(A) selection in more detail (S1A Fig). We were surprised to see that, according to both cluster (Fig 3, S2 Table sheet 3) and enrichment analyses (S2 Table sheet 2), mRNAs encoding protein kinases (Fig 3A) and RNA-binding proteins (Fig 3B) were selectively lost after poly(A) selection. Readers can analyse this themselves using the cluster viewer which is in the Supplement. By Northern blotting, we confirmed this result for two RNA binding protein mRNAs, *ZC3H32* and *ZC3H8* (S2A Fig).

**Fig 4:**
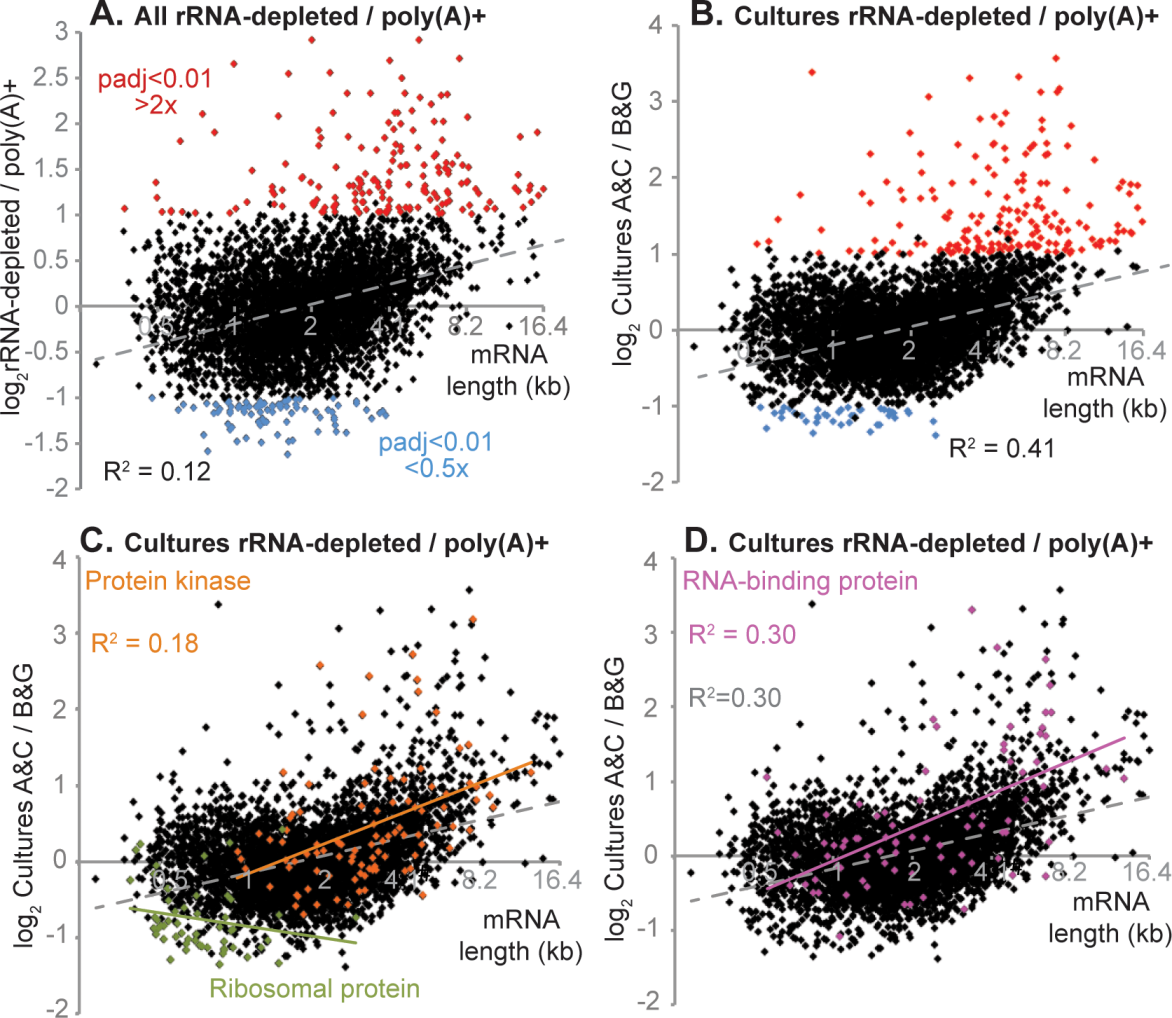
Poly(A) selection and rRNA depletion: effect of mRNA length. Different datasets were compared using DeSeq2 (S2 Table Sheet 1). The ratios of rRNA-depleted divided by poly(A)+ are shown on the y axis, and the mRNA length on the x-axis. Log-transformed values were used for the graphs and the regression analysis, but for clarity, the mRNA length axis has been labelled with the non log-transformed values. A. Results for all pooled datasets. Differences in RNA abundance were classed as significant if the adjusted p-value was less than 0.01, and the magnitude of the difference was at least 2-fold [64]. Correlation coefficients were calculated by Microsoft Excel. B. Results for selected cultures (the ones with values for most full genes). C. As (B), but with mRNAs encoding protein kinases in orange and mRNAs encoding ribosomal proteins in green. D. As (B), but with mRNAs encoding RNA-binding proteins in pink.

Loss of these particular functional sets might have been meaningful—perhaps they have very short poly(A) tails. On the other hand, many of the most affected protein kinase and RNA-binding protein mRNAs are rather long, either because of long open reading frames, or long 3'-untranslated regions (3'-UTRs) (S3 Fig). We therefore wondered whether poly(A) selection was resulting in the loss of long mRNAs. A comparison using all available datasets (Fig 4A, S2 Table) confirmed this suspicion, with particularly strong effects above about 4kb (2^12^ on the graph). Oddly, when we divided the datasets according to how the parasites had been grown, we found a clear length effect for RNA from cultured parasites (Fig 4B) but not RNA from rat blood (S2B Fig). However the poly(A)+ rat blood samples were biologically much more diverse and less well characterized than the cultures, so inter-sample variation might conceal a length effect. We therefore decided to follow up the result for cultures.

There are two obvious technical reasons why long mRNAs might get lost during poly(A) selection. One is degradation. Indeed, it has previously been demonstrated that poly(A) selection can result in preferential loss of sequence towards the mRNA 5'-end [33]. We counted only reads from open reading frames, so any mRNAs that were broken in the 3'-UTR would fail to be counted. Somewhat unexpectedly, there was no correlation between poly(A)+/ribo-minus ratios and the annotated 3'-UTR length (S2C Fig). This conclusion must however be regarded with caution because 3'-UTR lengths in the database are sometimes too short.

Further investigation suggested that it was indeed mRNA length that was important, rather than the functional class of the encoded protein. For mRNAs encoding both cytoskeletal proteins and protein kinases, the correlation between abundance and length was greater for the total mRNA length than it was for the open reading frame alone (Fig 4C; S2D–S2F Fig). Moreover, scrutiny of published RNASeq read density maps (TritrypDB) for several of the outliers among protein kinase mRNAs suggested that the annotated 3'-UTR lengths were incorrect. Similar length-abundance correlations were seen for mRNAs encoding RNA-binding proteins (Fig 4E), cell cycle proteins (S2G Fig) and translation factors (S2H Fig). There was less correlation for transporters (S2I Fig) and none for the relatively short mRNAs encoding ribosomal proteins (Fig 4C).

### Reporter experiments confirm that the length of an mRNA can affect its abundance after poly(A) selection

**Fig 5:**
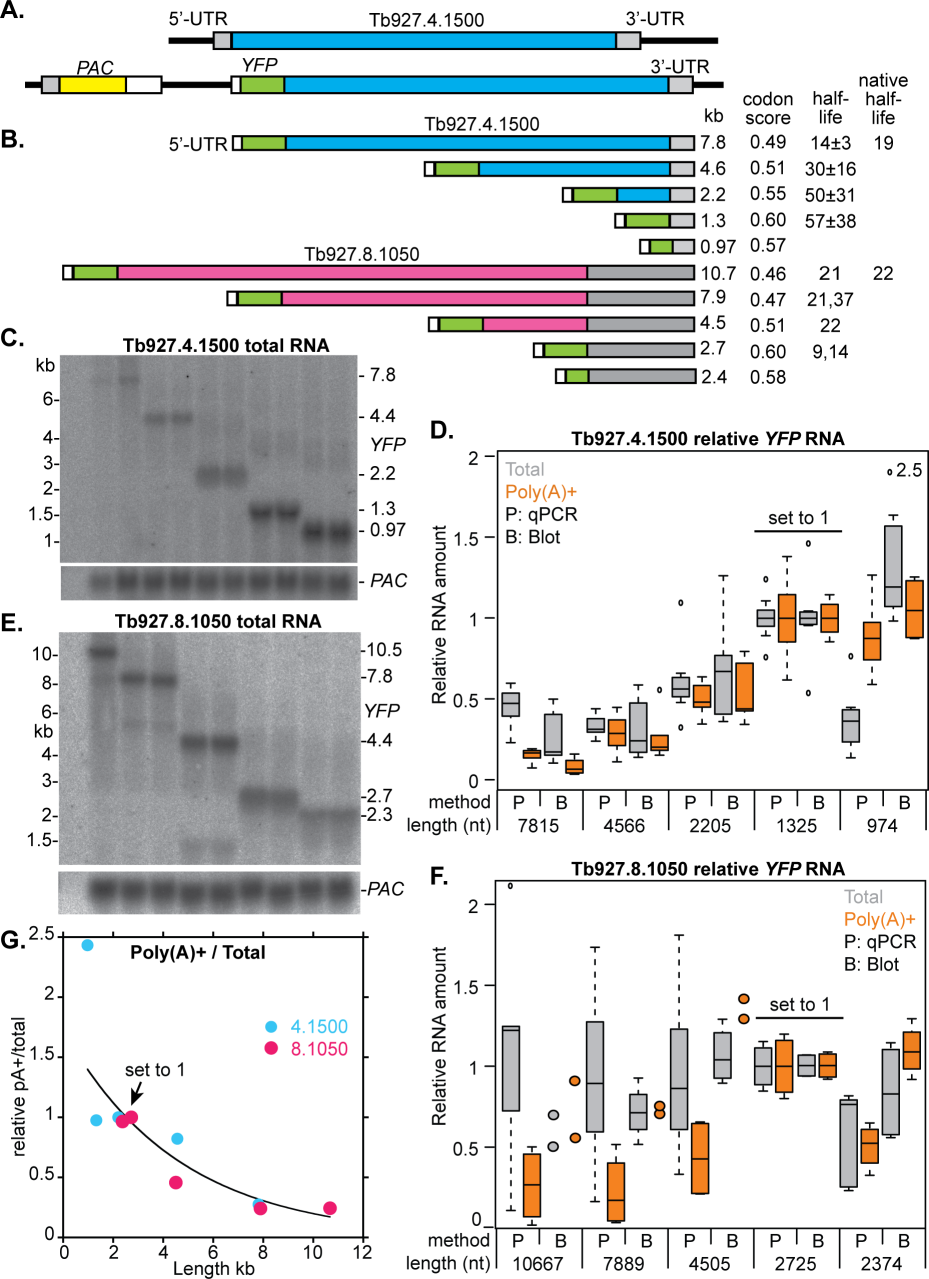
Reporter experiments: Effect of mRNA length on poly(A) selection and mRNA abundance. A. Cartoon of an intact Tb927.4.1500 locus (above) and a locus with integrated *PAC-GFP* (not to scale). B. Structures of *GFP* fusion mRNAs (to scale) with lengths. The codon optimality scores are calculated using a formula provided by Prof. M. Carrington (Cambridge University, personal communication). Approximate half-lives were estimated by real-time PCR of mRNA prepared 30 min after addition of Actinomycin D; each clone was measured once. C. Sample Northern blot for Tb927.4.1500; other blots are in S4D Fig. Amounts of *GFP* signal or PCR product relative to the result for the 1.3 kb mRNA. The boxes indicate the median value with 25th and 75th percentiles; whiskers extend to the most extreme data point that is no more than 1.5 times the length of the box away from the box. Tiny circles are outliers. E. Sample Northern blot for Tb927.8.1050; other blots are in S3F Fig. Amounts of *GFP* signal or PCR product relative to the result for the 2.7 kb mRNA. If less than 4 measurements were available, individual results are shown as coloured circles. G. Ratio of poly(A)+ to total mRNA. To make the values for the two genes comparable, the abundances of the Tb927.4.1500 reporter mRNAs were calculated relative to the 2.2 kb mRNA. (This results in exclusion of one dataset that lacks a value for that length.) The average relative mRNA abundance in poly(A)+ was divided by the relative abundance in total RNA. Results using medians were similar. The exponential curve was calculated in Kaleidograph using the combined data from both genes.

To investigate the effect of mRNA length directly, we experimentally changed the lengths of two open reading frames (Tb927.4.1500 and Tb927.8.1050), by integration of a yellow fluorescent protein (*YFP*) open reading frame at various positions relative to the endogenous start codon. This generated progressively shorter mRNAs (Fig 5A and 5B). The *YFP* mRNAs were measured by Northern blotting (Fig 5C and 5E, and S4 Fig) and by reverse transcription followed by real time PCR (qRT-PCR). The mRNA from the upstream puromycin resistance cassette (*PAC*) (Fig 5A) was used as a loading control. For both genes, the mRNA sizes were as expected (Fig 5C and 5E), but for Tb927.8.1050 there was also a shorter mRNA species (Fig 5F), which was roughly 20% of the total irrespective of length. Up to a length of 8 kb, results from blots and qRT-PCR were comparable (Fig 5D and 5F). The only exception was for the shortest mRNAs, which had a truncated *GFP* ORF; for these, results from qPCR were anomalous.

The abundance of total mRNA from the Tb927.4.1500 reporters decreased with increasing length (Fig 5C and 5D). To find out whether this might be due to differing half-lives, we measured mRNA abundance by qRT-PCR 30min after inhibition of splicing and transcription. Reassuringly, the half-life of the full-length fusion mRNA was similar to that of the unmodified version. However, the half-life increased with progressive truncations (Fig 5B). Thus for the Tb927.4.1500 locus, the increase in mRNA abundance with decreasing length can probably largely be attributed to increased mRNA stability. In contrast, for Tb927.8.1050 no reproducible length effect was seen on abundance (Fig 5E and 5F) and this was also the case for preliminary half-life measurements. As in other organisms [34], codon optimality can affect trypanosome mRNA half-lives (M. Carrington, Cambridge University, personal communication). This does not explain the difference between the two loci: for both, deletions of the open reading frame by *GFP* integration resulted in progressive increases in codon optimality (Fig 5B). Perhaps there are differences in codon distribution: clusters of non-optimal codons might have a bigger effect on translation than non-optimal codons that are spread uniformly throughout the sequence, and the positions of non-optimal codons relative to the start codon are known to be important [34].

Contrary to our previous conclusions from modeling [23, 35], results for these two genes did not yield any evidence for an effect of mRNA length that was independent of the half-life. More accurate half-life measurements would be needed to confirm this.

In the whole transcriptome analysis 60% of the mRNAs that were significantly depleted after poly(A) selection were longer than 4kb. Indeed, for the YFP fusion mRNAs, poly(A) selection caused loss of reporter mRNAs longer than 5 kb, and there was also some loss of the 4.5 kb Tb927.8.1050 locus mRNA (Fig 5D, 5F and 5G). We concluded that poly(A) selection can cause loss of mRNAs longer than 4 kb, but also that losses are variable. Perhaps other sequence characteristics also contribute to the mRNA yield.

### Differences between blood and CSF trypanosomes

**Fig 6:**
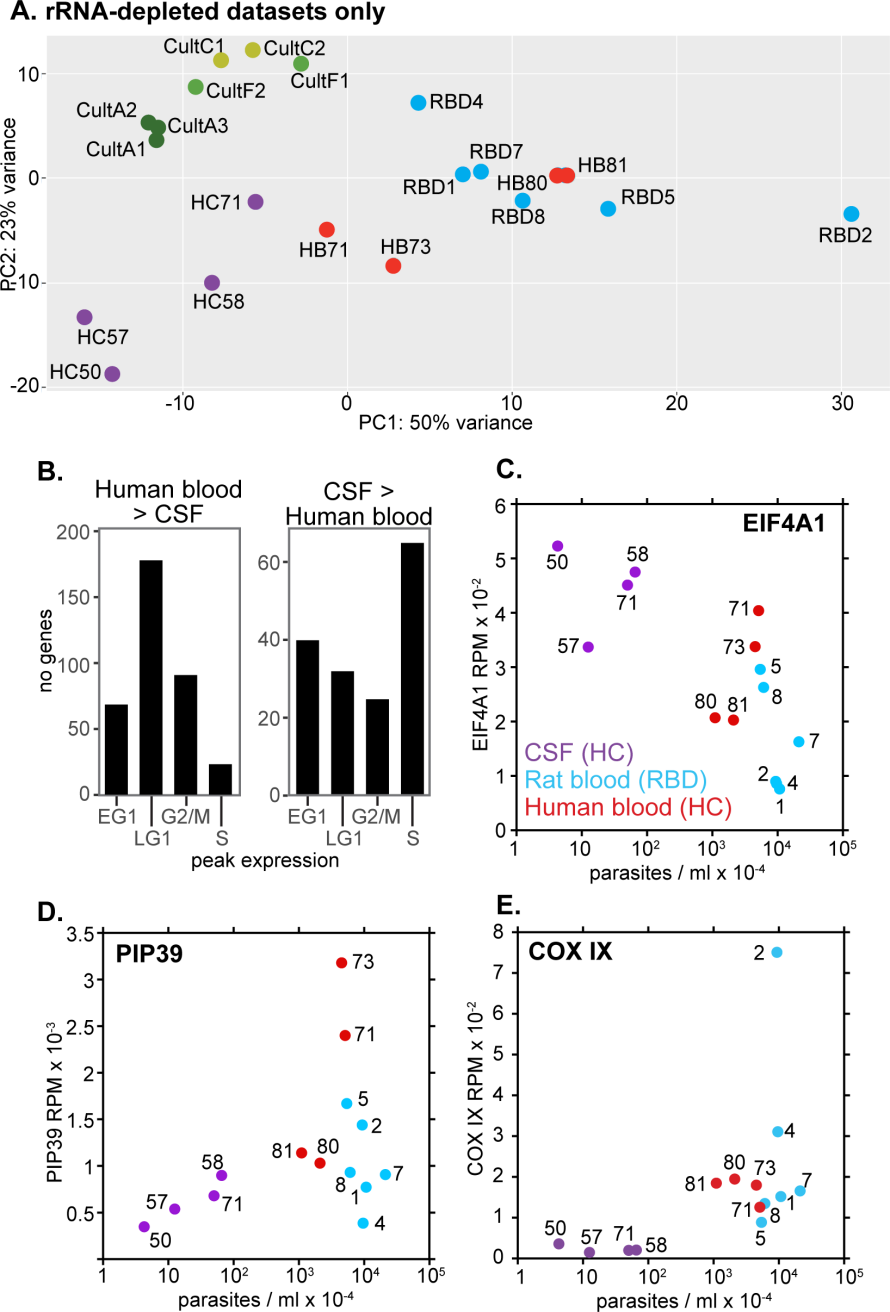
Relationships between cell density and expression of chosen genes. A. Principal component analysis including blood and CSF RNAs prepared using a common protocol, and for rRNA-depleted RNA from culture. B. The mRNAs that were significantly different between human blood and CSF were categorised according to whether they show peak expression in a particular cell cycle stage. RNAs that show no cell cycle regulation are not included. C. Expression of EIF4A mRNA (RPM) relative to cell density (log scale). The key is on the blot and sample numbers are indicated. Note that the densities for the human blood samples are approximate because they were estimated from stained thin films (see methods). D. As (C), but showing *PIP39* mRNA. E. As (C), but showing *COX IX* mRNA. Additional results are in S6 Fig.

In subsequent comparisons, we considered only the datasets derived from rRNA-depleted RNA. The principal component analysis for these samples (Fig 6A) suggested that from the trypanosomes' point of view, there are few differences between rat and human blood; the total number of mRNAs with significantly different abundance, 125, was very low (S2 Table) and probably not far from random variation. The CSF transcriptomes were separated from those for blood, but it was notable that our only samples from the CSF and blood of a single patient (C71 and B71) were relatively similar. Notably, the CSF parasite transcriptomes more closely resembled those of log-phase cultured cells (Fig 6A) than did those of blood parasites. Differences between the various samples could be due to differences in available nutrients or immune responses, but most obviously, from the presence of stumpy forms, since densities in blood were much higher than in CSF.

Cluster analysis of the samples from humans and rats only (S5 Fig, S3 Table) distinguished two groups. One group included the CSF samples and two human blood samples (HB71 and HB73), and the other group included the remaining blood samples. The former group showed higher expression of mRNAs encoding cytoskeletal proteins, several translation factors, tRNA charging enzymes, RNA degradation pathway proteins, and some protein kinases (clusters 3 and 16, S3 Table). At the same time, it showed lower expression of mRNAs encoding numerous mitochondrial proteins (clusters 12 and 18). Comparison of the human blood and CSF parasite transcriptomes (S2 Table, sheet 1) revealed 320 mRNAs that were lower in blood, and these were again significantly enriched in mRNAs encoding cytoskeletal proteins (S2 Table, sheet 2). 830 mRNAs were higher in blood, with enrichment for mitochondrial electron transport and amino acid transport (S2 Table, sheet 2). Cell-cycle-regulated genes that were over-expressed in blood mainly show peak expression in G1, whereas many of those that were more abundant in CSF peak in S-phase (Fig 6B) [36]. All of these results suggested that the CSF parasite population included more actively multiplying parasites than the bloodstream populations. Expression of mitochondrial proteins suggested that that the bloodstream populations included some parasites that were beginning to differentiate to stumpy forms.

To examine the link between gene expression and cell density, we looked at a few examples. Results for the translation initiation factor EIF4A, the stumpy-inducing phosphatase PIP39 and a cytochrome oxidase subunit (Fig 6C–6E), as well as various other regulated mRNAs (S5 Fig) revealed no simple relationship between expression and cell density. For the rat blood samples, there was also no correlation with attaining the plateau of parasitaemia. This was consistent with our previous morphological analysis of the rat samples (Fig 1B).

### Comparison between trypanosomes from culture and from humans

Finally, we looked at the differences between cultured and human parasites. We focus here mainly on the CSF parasites: differences between culture and blood were more difficult to interpret due to the likely presence of growth-arrested parasites in the bloodstream (see above). The results suggested that there are indeed some differences which must be considered when using cultured parasites as a model.

Genes that were more highly expressed in the human CSF samples included those encoding four membrane proteins; but these were mainly from multi-gene families, which can vary between strains. The products of CSF up-regulated mRNAs were also enriched for ribosomal proteins (S2 Table sheet 2). More interestingly, the increased mRNAs encoded nine protein kinases, and three potential cyclins, two of which (CYC10 and CYC11) were also increased in human blood (S2 Table sheet 1). The mRNAs in rat blood and cultured trypanosomes were previously compared in a ribosomal profiling study [22]. There was no significant correlation between those results and ours. Nevertheless, in that study too, the blood parasites had higher levels of mRNAs encoding CYC10 and CYC11, various protein kinases, protein phosphatases, and RNA-binding proteins (S2 Fig Sheet 1).

Cultured trypanosomes had higher mRNA levels than CSF trypanosomes for mRNAs encoding half of the Sm complex and some other splicing factors; various RNA-binding proteins including ZFP2 and ZFP3; 26 cytoskeletal proteins; 51 mitochondrial proteins, 26 proteins involved in vesicular transport, numerous translation initiation factors, and the whole of the core proteasome. The differences for blood trypanosomes were to some extent similar, but in this case the cultures also expressed more translation initiation factor mRNA. These results might mean that the cultured cells are multiplying faster than the cells in the patients.

**Fig 7:**
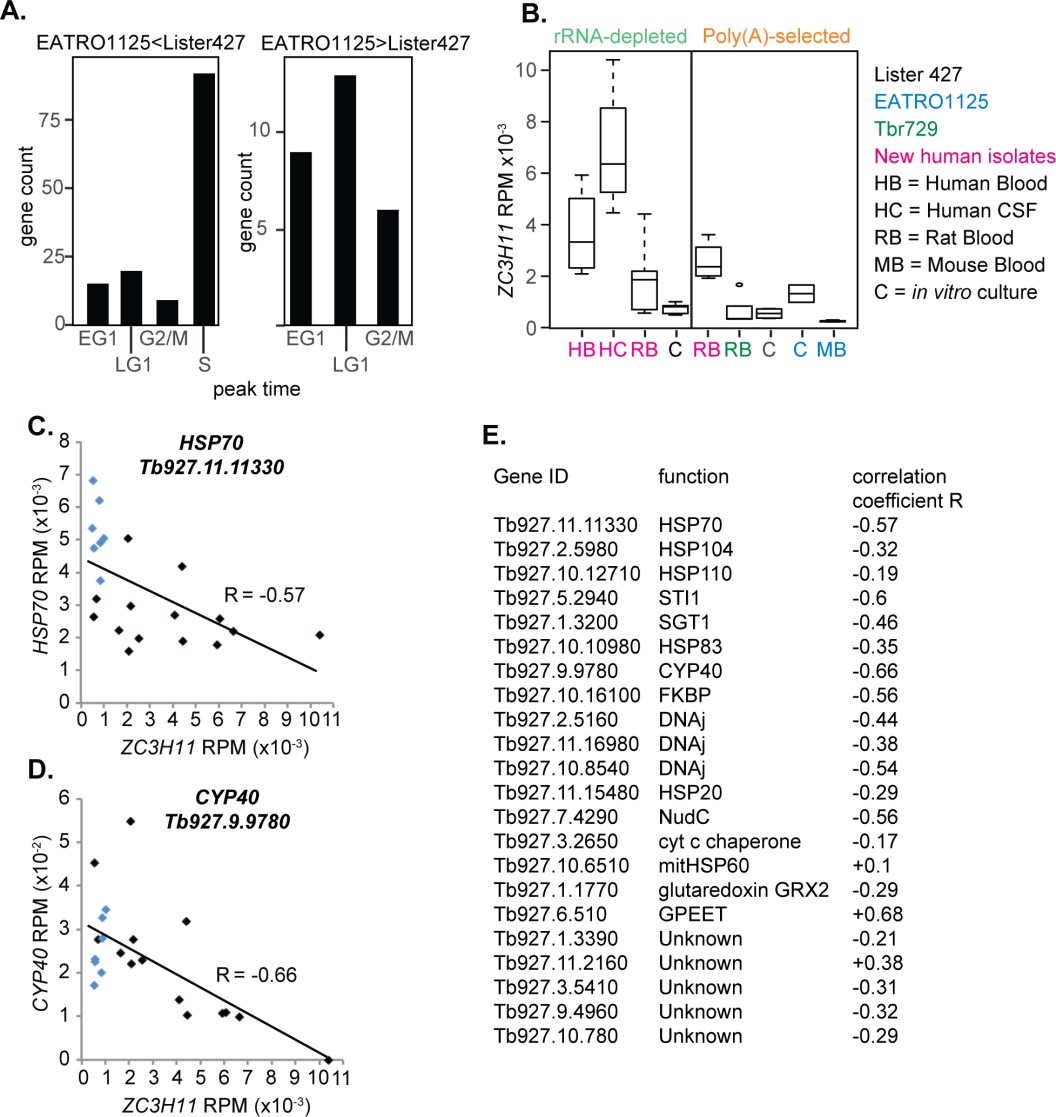
Differences between trypanosomes from animals and culture. A. The mRNAs that were significantly different between EATRO1125 and Lister 427 cultures (poly(A)+ datasets) were categorized according to whether they show peak expression in a particular cell cycle stage. RNAs that show no cell cycle regulation are not included. B. Levels of *ZC3H11* mRNA in different types of trypanosome sample. C. Relationship between *ZC3H11* and *HSP70* mRNA amounts. D. Relationship between *ZC3H11* and *CYP40* mRNA amounts. E. Pearson correlation coefficients for published ZC3H11 targets [37].

Since all of the ribo- culture datasets were from monomorphic trypanosomes, we also compared poly(A)+ mRNAs from pleomorphic and monomorphic cultures. This revealed that cultured pleomorphic EATRO1125 had lower expression of numerous RNA-binding proteins, and some RNA decay and cytoskeletal proteins (S2 Table Sheet 1), than cultured monomorphic Lister 427. Cell cycle analysis (Fig 7A) suggests that this reflects more active division of the Lister 427 cultures. Unsurprisingly, this indicates that pleomorphic cultured cells ought to resemble parasites in patients more closely than monomorphic cells do.

### Comparison between trypanosomes from culture and from humans: The stress response and ZC3H11

The zinc finger protein ZC3H11 is a positive regulator of mRNAs encoding protein refolding chaperone complexes [37], and is required for the survival of procyclic forms after heat shock. After a brief heat shock, *ZC3H11* mRNA is unaffected but its translation is strongly induced [38]. Remarkably, in all of our new ribo-minus transcriptomes, *ZC3H11* was among the ten most abundant mRNAs, being 4–6 times more abundant than in cultured trypanosomes. Comparison of all the datasets also revealed strain differences: *ZC3H11* mRNA levels were lower in culture-adapted parasites than in the newly-isolated ones (Fig 7B). This is definitely a difference in regulation: *ZC3H11* is a single-copy gene in the new rat blood trypanosomes as well as in Lister 427 [17]. All of the human patients from our study had fever so it may be that the *ZC3H11* mRNA is stabilized by prolonged elevated temperatures. Paradoxically, though, many chaperone mRNAs were significantly *lower* in the CSF parasites than in culture, and some known ZC3H11 target mRNAs [37] showed a weak *inverse* correlation with *ZC3H11* mRNA (Fig 7C–7E). Further comparison revealed over 200 mRNAs with expression that was either negatively or positively correlated with ZC3H11 mRNA in both ribo-minus and poly(A)+ datasets (S2 Table Sheet 4). Products of negatively correlated mRNAs included cytoskeletal proteins, protein kinases and phosphatases, while for positively correlated mRNAs there were some mitochondrial and ribosomal proteins. The meaning of these results is unclear, since the level of ZC3H11 protein does not correlate with the mRNA [39]. Contrary to the results shown here, in the Jensen ribosome foot-printing study, *ZC3H11* mRNA was lower in blood than in culture parasites—but the blood parasites yielded 10 times more ribosome footprints [22]. ZC3H11 is also phosphorylated, which might affect its activity [37, 39].

### Conclusions

This is, to our knowledge, the first study that compares transcriptomes of parasites from several different labs, with different strains, growth conditions, and RNA preparation methods. We discovered that each of these affects the transcriptome.

It was already known that poly(A) selection and rRNA depletion affect RNA-Seq-derived trypanosome transcriptomes [40], and such effects have been comprehensively demonstrated for mRNAs for other species, including humans [41, 42]. From analysis of the data, combined with reporter experiments, we concluded that technical factors, such as trapping of RNA in the matrix, strongly contribute to depletion of long mRNAs. The reason that the differences are concentrated within mRNAs encoding particular functional protein classes may be that these classes have a disproportionate number of long mRNAs; in the case of both protein kinases and RNA-binding proteins, this is because their 3'-UTRs are longer than average (S3 Fig). mRNAs encoding ribosomal proteins are, in contrast, unusually short (S3 Fig). Since the 3'-UTR annotations are not all correct, the extent to which other factors contribute is not certain. Some mRNAs may not be retained on the oligo d(T) matrix because they have very short poly(A) tails. Although poly(A) tails usually protect from degradation and promote translation, well-expressed Opisthokont mRNAs tend to have short tails [43]. We compared mRNA half-lives [35] and ribosome densities [22, 23], but for these characteristics we found no significant difference between the trypanosome mRNAs that were enriched or depleted by poly(A) selection.

When we started this study, we expected that of the available laboratory models, trypanosomes growing in rodent blood would have transcriptomes that most closely resembled those of pleomorphic trypanosomes growing in humans. Our results confirmed this expectation, but also revealed some intriguing differences between trypanosomes growing in different environments. Several hundred mRNAs were significantly different between cultured and human-grown trypanosomes. The functions of the proteins encoded by those mRNAs suggested that the cultured parasites might be multiplying faster than parasites in blood, and that the blood parasites were affected by environmental stresses. Although some of the differences in gene expression might have been due to the growth environments, others can probably be attributed to culture adaptation of the parasites. All of the data from cultures were for Lister 427 parasites, which were probably originally isolated from a cow in Tanganyika (now Tanzania) (see http://tryps.rockefeller.edu/DocumentsGlobal/lineage_Lister427.pdf). The cells have been serially passaged for many years, and in culture since about 1985. To understand the effects of culture adaptation it will be necessary to follow parasite genomes and transcriptomes during that process.

There was no systematic correlation between human parasitaemias and expression of mRNAs that are increased in stumpy forms. In rats, in the samples analysed, there was also no correlation between parasitaemia and parasite morphology. Stumpy forms of *T*. *gambiense* were originally reported in the descending phase of human parasitaemia [44]; perhaps some of the patients were also in that phase. Moreover, some tissues may harbour higher trypanosome densities than are present in the blood, and thus accumulate stumpy induction factor. The resulting stumpy parasites might subsequently escape into the circulation. Previous rodent studies did not support this idea [6, 7, 45, 46]. However, recent results do suggest that tissue and blood parasitaemias may be different. *T*. *b*. *gambiense* were found in the skin of asymptomatic humans lacking detectable blood parasitaemia [47], and relatively high proportions of stumpy-form (PAD1-positive) *T*. *brucei* were detected in mouse skin and adipose tissues [47, 48].

An important motivation for our study was to find out whether CSF trypanosomes are significantly less metabolically active than those in blood, and thus less susceptible to drug treatment. Overall, if transcriptomes can be taken as a guide to enzyme expression, the results did not provide evidence for systemic metabolic differences between blood and CSF trypanosomes. If anything, the CSF parasites are likely to be more metabolically active than those in the blood, and thus more susceptible to any drug that targets parasite metabolism or multiplication.

## Methods

### Ethics statement

For the human studies, ethical approval of protocols was obtained from the Ministry of Health and Uganda National Council of Science and Technology (Ethical approval No. HS 729), Uganda, and the ethics committee of University of Heidelberg, Germany. All patients recruited into this study received written and verbal information explaining the purpose of the study and they gave informed consent. The ethical consent forms were written in English and translated into the local languages. For the children and adolescent participants (below 18 years), parents or guardians gave informed consent on their behalf.

Animal experiments in this work were carried out in accordance with the local ethical approval requirements of the University of Edinburgh and the UK Home Office Animal (Scientific Procedures) Act (1986) under license number 60/4373, or in Makerere University with approval of the College of Veterinary Medicine Animal Resources and Biosecurity research and ethics committee, with approval number SBLS/REC/16/137b.

### Human sample collection

Samples were collected as described previously [17] during routine sleeping sickness diagnosis at Lwala hospital in the Kaberamaido district of North-Eastern Uganda. In order to confirm that all the cases were *T*. *b*. *rhodesiense* infections, PCR was carried out on the *SRA* gene as described in [49]. Up until sample 60, both blood and CSF samples were centrifuged to obtain a cell pellet for CSF, and a buffy coat for the infected blood. These were either resuspended directly into Trizol, then frozen in liquid nitrogen, or the cells were stored in liquid nitrogen for later RNA extraction. For samples 61 onwards, both CSF and blood were placed directly into PAXgene tubes. For both PAXgene and Trizol, RNA was prepared according to the manufacturer's instructions.

CSF cell counts were made directly from undiluted samples. For blood, cell counts were estimated at the clinic using thin smears stained with Giemsa. To convert these values to parasitaemias, we used blood from infected rats, counting parasites in diluted samples in a haemocytometer, and counting parasites from the same samples on dried smears. The results for human parasitaemias are therefore only approximations.

### RNA sequencing for human blood and CNS samples, and rat blood samples

Human RNA samples were initially checked for their integrity on the Agilent Bioanalyzer 2100 (Agilent RNA Nano 6000 kit, 5067–1511). The human blood samples showed considerable degradation. All samples (blood and spinal fluid) were prepared for sequencing using the Illumina TruSeq Total Stranded RNA preparation kit (Illumina, RS-122-2301). Between 75–750 ng total RNA was used as input material. rRNA depletion was performed on the samples dependent on their origin; those from blood were depleted with RiboGlobin (Illumina), those from the spinal fluid with Ribo-Gold (H/M/R) (Illumina). Since these kits are optimised for depletion of mammalian rRNA, most trypanosome rRNA remained in the sample. Due to the degradation of the samples, the binding time for depletion was increased to 5 minutes, and the subsequent fragmentation time was decreased from the normal 8 minutes to 3 minutes. PCR cycles were decreased from the recommended 15 to 13 cycles for the human samples. All human samples were processed with the same batches of Paxgene tubes and reagents for RNA handling and library preparation. Different batches were used for the rat samples.

The finished libraries were equimolar pooled and sequenced with the Illumina NextSeq500 System, at the EMBL Genomics Core Facility, where 75 Single-end reads were generated (Illumina, FC-404-2005). The raw data are available at Array express under accession numbers E-MTAB-5293 and E-MTAB-5294 (human) and E-MTAB-6125 (rat). The rat samples were sequenced 1–2 years after the human samples.

### RNA sequencing for mouse long slender trypanosomes

Trypanosomes were purified on DEAE cellulose, then RNA was isolated using RNeasy column purification (Qiagen) with on-column DNase treatment according the the manufacturer's instructions. Total RNA samples were subjected to oligo(dT)-selection and paired-end sequencing at the Beijing Genomics Institute.

### RNA-Seq read counting

RNAseq datasets were retrieved as FASTQ files. In case of paired-end data sets (MB_A, MB_B, MAd), only one end per sample was analysed to ensure comparable results with single-end data. All sets were processed as follows: First, the overall read quality was investigated using FastQC [50]. Hereby, overrepresented sequences were identified, making up more than 0.1% of all reads in a set. Since this overrepresentation is not expected in a standard RNAseq experiment, and in our experience these sequences have often rRNA origin, they were removed using Cutadapt [51]. The resulting cleaned reads were then aligned to the *T*. *brucei* TREU927 genome (release 9.0) using bowtie 2 [52] with a maximum mismatch count of one and each read was allowed to align to the genome up to 20 times. This helps in making sure that each read originating in a multi-gene family, aligns in each member, which is necessary for later subsetting for a unique gene list [29]. The alignment was used for read counting, and utilizing a custom script which is based on Samtools [53] to count the number of aligned reads within each annotated coding sequence. The whole process was automated using a python based pipeline [25].

### Statistical analysis of RNA-Seq data

For statistical analysis we used a unique gene list which holds single representatives for each multi-gene family, and unique genes (total about 7000 genes) [29]. All analysed data sets were combined in one read count table. Genes for which no data could be retrieved in some samples (NA-values), were removed, and the rest was analysed using the R package DESeq2 [31, 54, 55]. The DESeq2 experimental design included only the affiliation of each sample to the original data set. The significance level alpha was set to 0.01. Heat maps for overall comparison and co-regulation studies were generated using the rlog function of DESeq2. The rlog function transformed the read counts and normalized the data to the sequencing depth and also shrank the effect size of genes with low read counts to prevent overestimation. The rlog transformed counts were then given to the pheatmap [56] package which was instructed to generate previously mentioned numbers of kmeans-clusters of all unique genes, to deduce the euclidean hierarchical clustering of the kmeans-clusters and the samples and to plot the final heat map. Each gene was annotated using a manually curated list (see Supplementary Tables). Class enrichment within clusters was done using Fisher's exact test. Occurrence of each gene class within the studied cluster and within the unique gene list (all genes were removed which had incomplete data in the read table) was identified and for each class a two dimensional contingency table was generated. Fisher's exact test p-value for overrepresentation was calculated and corrected using the Benjamaini-Hochberg method for multiple testing [57].

### Cloning, real-time PCR and Northern blotting

PAC-YFP cassettes were integrated into the genome after cloning of suitable fragments into the plasmid p2675 [58] to direct homologous recombination [59]. All oligonucleotides used are listed in S4 Table. Clones were checked by Northern blotting. Total RNA was made using the RNAeasy Midi kit (Qiagen) or peqGold Trifast (Paqlab). Poly(A)+ RNA was selected using the Qiagen Oligotex mRNA kit. After denaturing formaldehyde gel electrophoresis, the RNA was subject to limited depurination (0.25M HCl, 15 min) to ensure efficient transfer of longer mRNAs. Northern blots were hybridised with radioactive probes covering the whole *YFP* or *PAC* ORFs.

For quantitative PCR (RT-qPCR) reverse transcription was done using random hexamer primers, with Superscript IV at 50°C, 15 minutes and the qPCR was done using LightCycler 480 SYBR Green I Master mix (Roche) or Luna Universal qPCR Master Mix (NEB) using LightCycler 480 II, Roche. Melting curves were checked using 95°C 10 s, 4.8°C/s; 65°C 1 min., 2.5°C/s; 95°C 0.11°C/s. For the qPCR slightly different procedures were used. The protocol for LC480 master mix was; denaturation 95°C 1min., 4.8°C/s; 45 amplification cycles of 95°C 20 s, 4.8°C/s, hybridization 60°C 20 s, 2.5°C/s, elongation 72°C 7 s, 4.8°C/s, 45 cycle. For Luna-Master Mix hybridization was for 30 s, and we used 40 cycles. Signals or measurements for *YFP* were normalized to those from *PAC*, to allow for differences in input RNA and for possible copy number variation. Then, one of the shortest mRNAs was used as a standard to calculate relative mRNA amounts.

To estimate mRNA half-lives, we inhibited mRNA processing and transcription using sinefungin and Actinomycin D, and RNA was isolated 30 minutes later [40]. RNA from cells with and without inhibition was quantified by RT-qPCR.

## Supporting information

S1 Table All sample and sequence data. For legends see sheet 0.
(XLSX)

S2 Table DESeq2 results. For legends see sheet 0.
(XLSX)

S3 Table Cluster analyses for rat blood and human blood and CSF. For legends see sheet 0.
(XLSX)

S4 Table Oligonucleotides and details of cloning. (XLSX)

S1 Fig Principal component analysis for poly(A)+ and ribo-minus samples. (PDF)

S2 Fig Poly(A) selection and mRNA length. All graphs (panels B—I) show the DeSeq ratios for ribosomal-RNA-depleted RNA divided by poly(A)+ RNA.
A. The mRNAs encoding ZC3H32 (~10 kb), ZC3H8 (6.6 kb), and trypanothione synthetase (3.4 kb) were detected on Northern blots of 8 independent RNA samples. (This was re-hybridization of two of the blots shown in S3 Fig). The *ZC3H32* and *ZC3H8* signals were then divided by the trypanothione synthetase signal. The boxes indicate the median value with 25th and 75th percentiles; whiskers extend to the most extreme data point that is no more than 1.5 times the length of the box away from the box. Circles (not seen here) are outliers.
B. Rat blood samples
C. For cultures: relationship with 3'-UTR length
D. For cultures: relationship with coding region length, with results for cytoskeletal proteins superimposed.
E. For cultures: relationship with mRNA length, with results for cytoskeletal proteins superimposed.
F. For cultures: relationship with coding region length, with results for protein kinases superimposed.
G. For cultures: relationship with mRNA length, with results for proteins involved in the cell cycle superimposed.
H. For cultures: relationship with mRNA length, with results for translation factors superimposed.
I. For cultures: relationship with mRNA length, with results for transporters superimposed.
(PDF)

S3 Fig Box plots showing characteristics of mRNAs encoding proteins of different functional classes. The broken line indicates the median for all genes and the colours are the same as in Fig 4 and S2 Fig. No class was statistically significant (<0.05) from the others by ANOVA; even for ribosomal proteins, the adjusted p-value was 0.1.
(PDF)

S4 Fig Reporter gene expression. *GFP* Northern blots for Tb927.4.1500 (A) and Tb927.8.1050 (B).
(TIF)

S5 Fig Comparison of human and rat blood datasets for recently isolated *T*. *rhodesiense*. A. Principal component analysis.
B. Clustering of genes according to differences in expression. The genes in each cluster are in S3 Table. See the trypclusterviewer (S1 Folder) for details.
(PDF)

S6 Fig Relationship between density and gene expression. Extra panels like Fig 6, with different genes.
(PDF)

S1 Folder Cluster analysis for all ribo-minus and poly(A)+ datasets. (ZIP)

S1 Text VSG contigs for HB80. (FASTA)

S2 Text VSG contigs for HB81. (FASTA)

S1 app This is a zipped folder containing the ClusterViewer script, which runs in R or RStudio, with all poly(A)+ and ribo-minus expression values (from DESeq2) already loaded. It enables readers to examine the data themselves. Instructions for how to change the datasets and the comparisons are included.
(ZIP)
