# Supplementary figures and images for: Transcriptomes of Trypanosoma brucei rhodesiense from sleeping sickness patients, rodents and culture: Effects of strain, growth conditions and RNA preparation methods
Source: PLoS Negl Trop Dis. 2018 Feb 23;12(2):e0006280. doi: 10.1371/journal.pntd.0006280 (PMC5842037; doi:10.1371/journal.pntd.0006280)

# A. Ribominus and poly(A)+ datasets

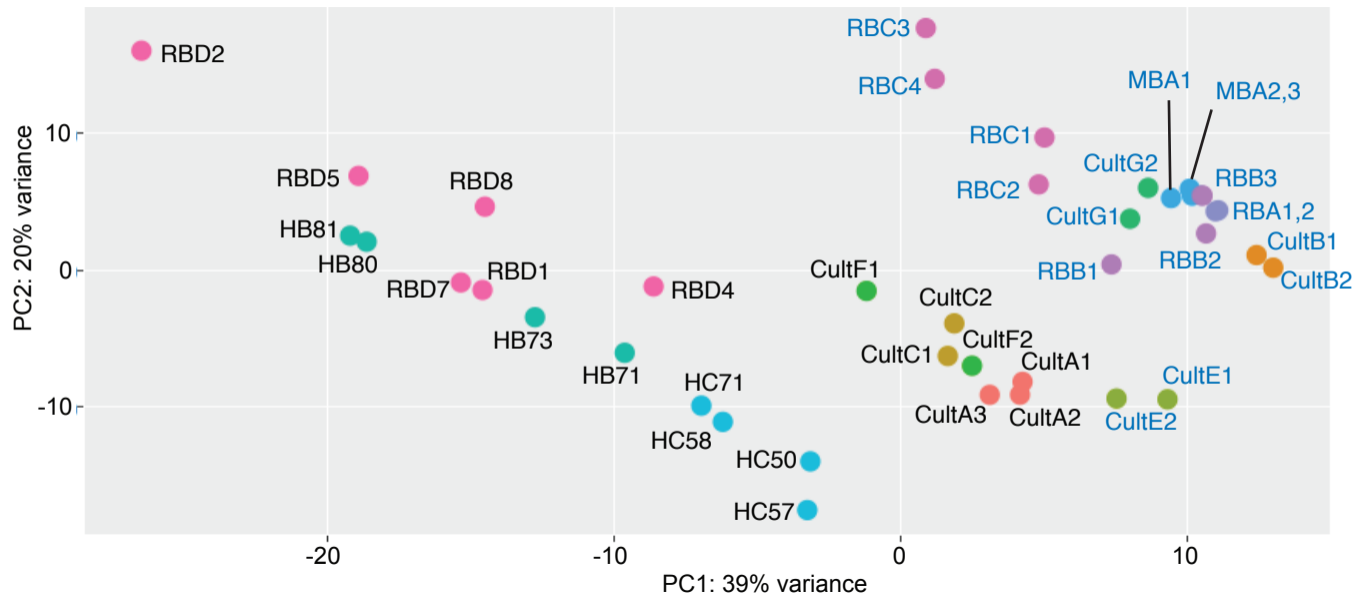

Supplement: S1 Fig — (PDF) [file pntd.0006280.s005.pdf]

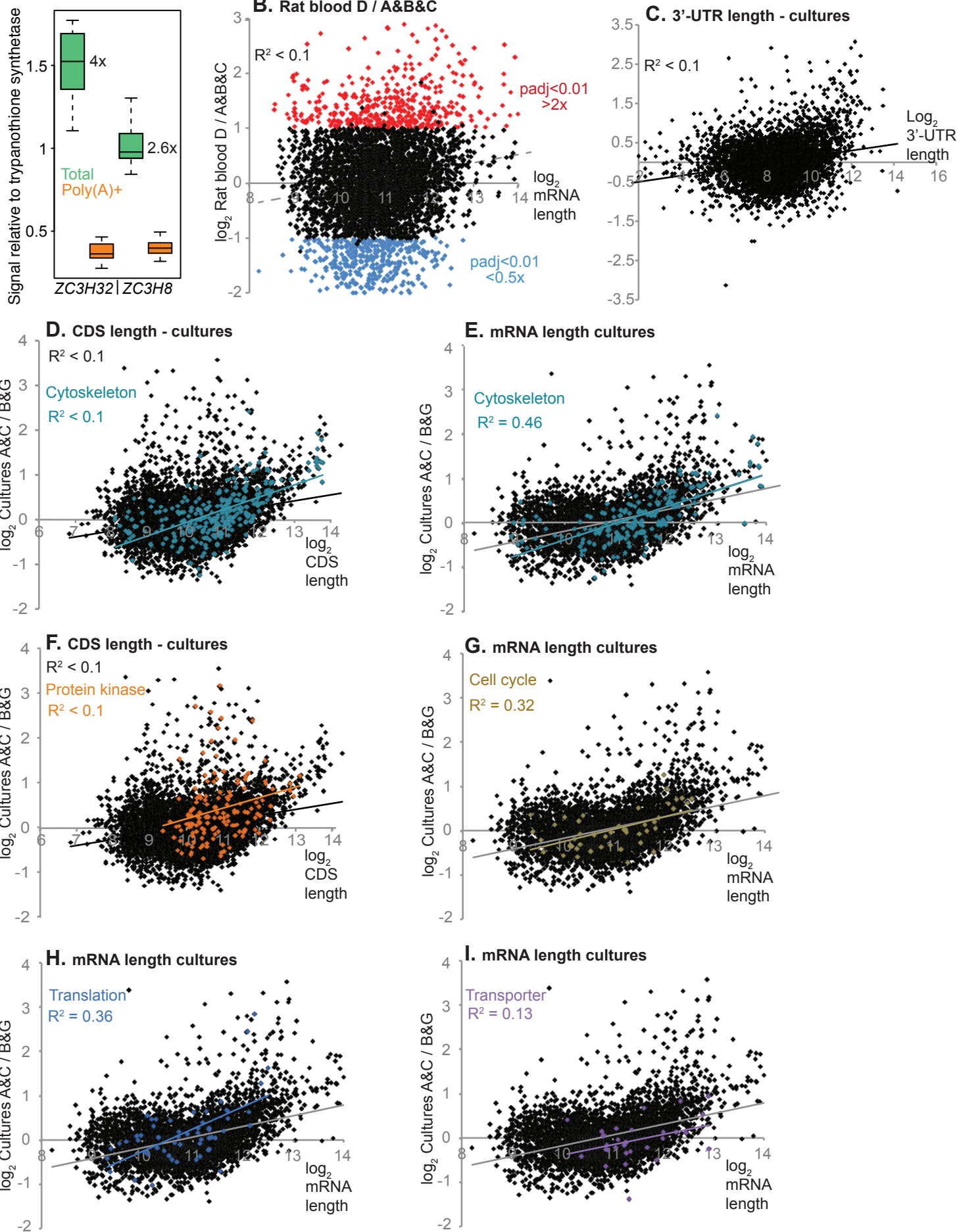

Supplement: S2 Fig — All graphs (panels B—I) show the DeSeq ratios for ribosomal-RNA-depleted RNA divided by poly(A)+ RNA. A. The mRNAs encoding ZC3H32 (~10 kb), ZC3H8 (6.6 kb), and trypanothione synthetase (3.4 kb) were detected on Northern blots of 8 independent RNA samples. (This was re-hybridization of two of the blots shown in S3 Fig). The ZC3H32 and ZC3H8 signals were then divided by the trypanothione synthetase signal. The boxes indicate the median value with 25th and 75th percentiles; whiskers extend to the most extreme data point that is no more than 1.5 times the length of the box away from the box. Circles (not seen here) are outliers. B. Rat blood samples C. For cultures: relationship with 3'-UTR length D. For cultures: relationship with coding region length, with results for cytoskeletal proteins superimposed. E. For cultures: relationship with mRNA length, with results for cytoskeletal proteins superimposed. F. For cultures: relationship with coding region length, with results for protein kinases superimposed. G. For cultures: relationship with mRNA length, with results for proteins involved in the cell cycle superimposed. H. For cultures: relationship with mRNA length, with results for translation factors superimposed. I. For cultures: relationship with mRNA length, with results for transporters superimposed. (PDF) [file pntd.0006280.s006.pdf]

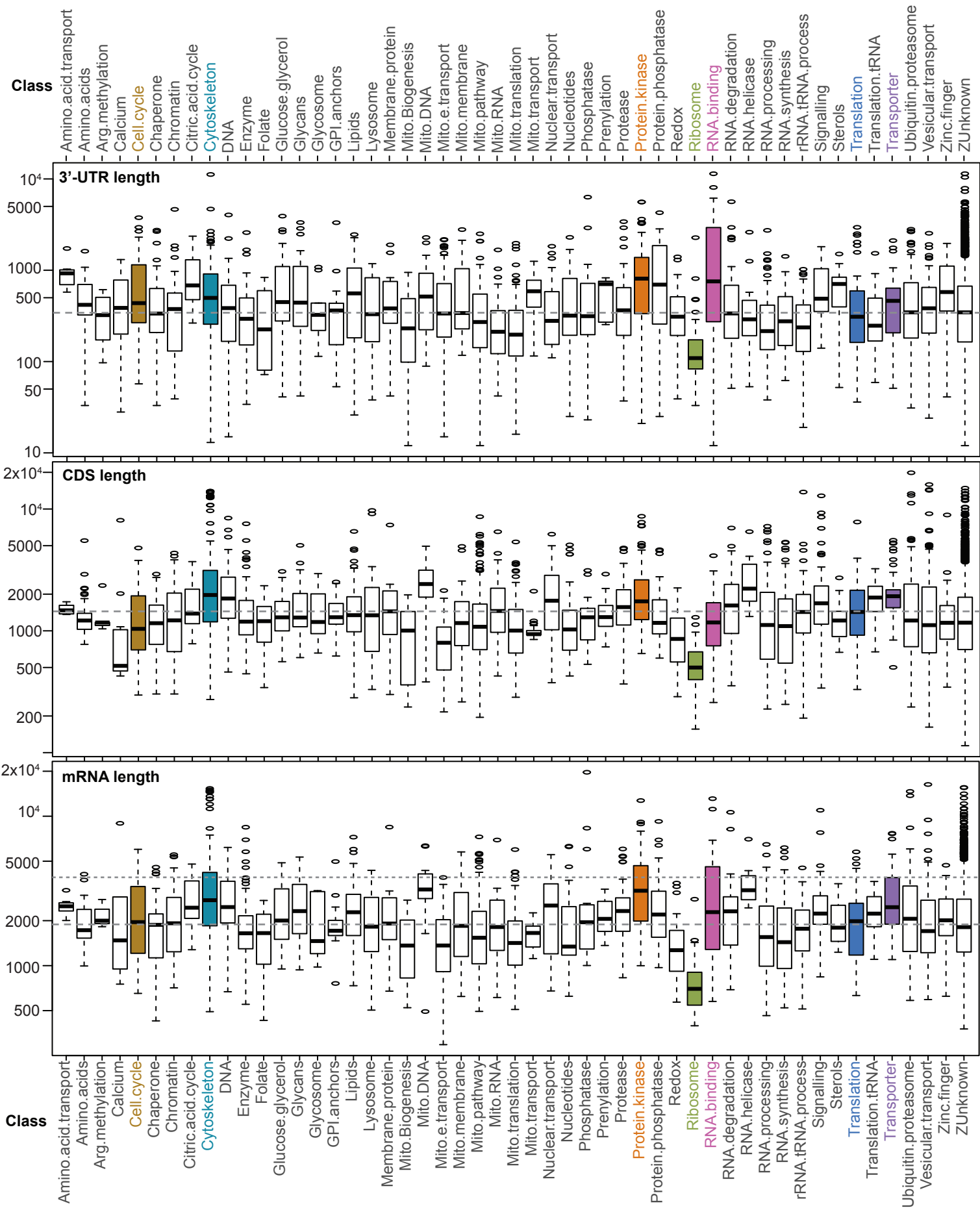

Supplement: S3 Fig — The broken line indicates the median for all genes and the colours are the same as in Fig 4 and S2 Fig. No class was statistically significant (<0.05) from the others by ANOVA; even for ribosomal proteins, the adjusted p-value was 0.1. (PDF) [file pntd.0006280.s007.pdf]

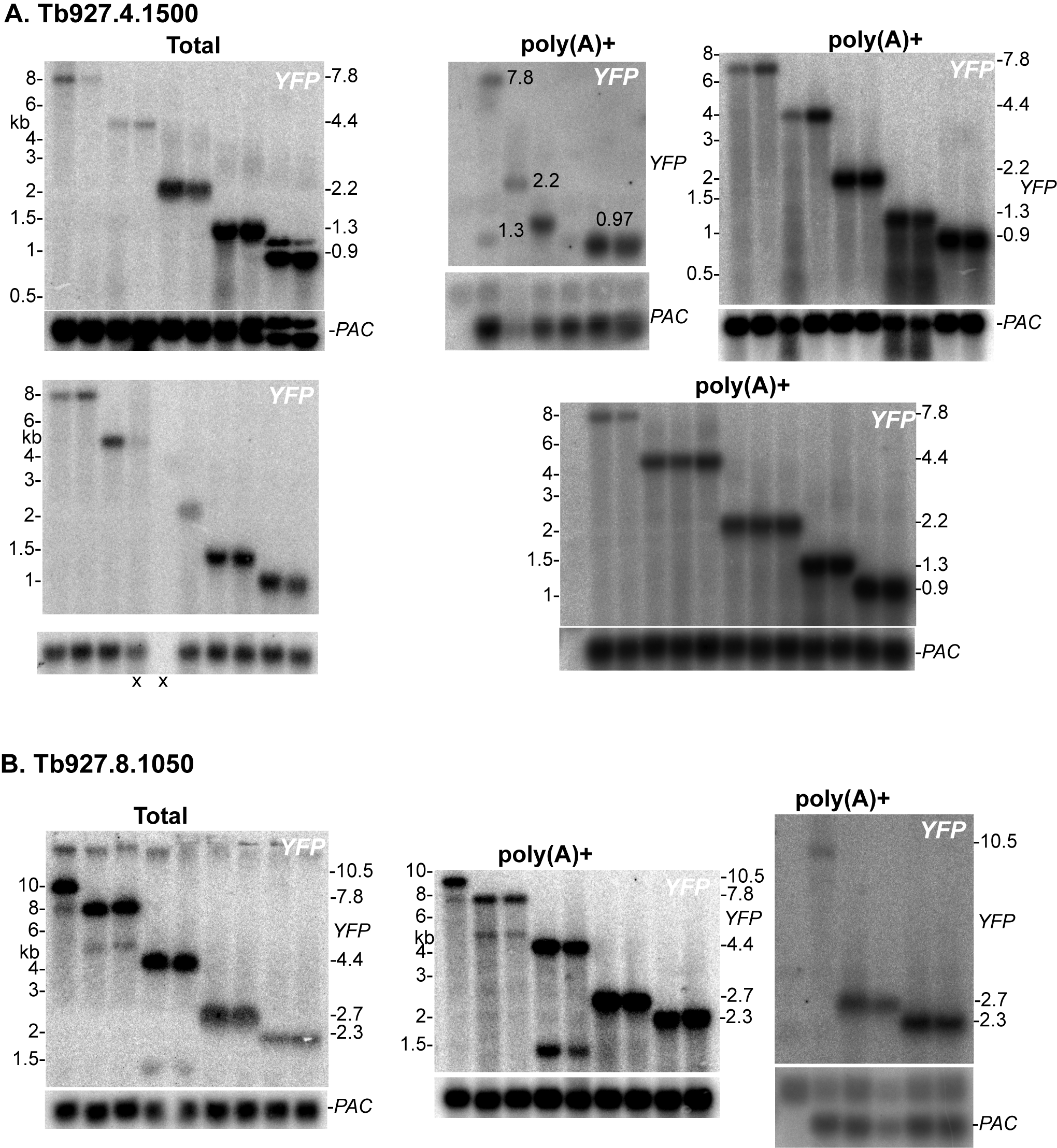

Supplement: S4 Fig — GFP Northern blots for Tb927.4.1500 (A) and Tb927.8.1050 (B). (TIF) [file pntd.0006280.s008.tif]

A. rRNA-depleted human and rat datasets

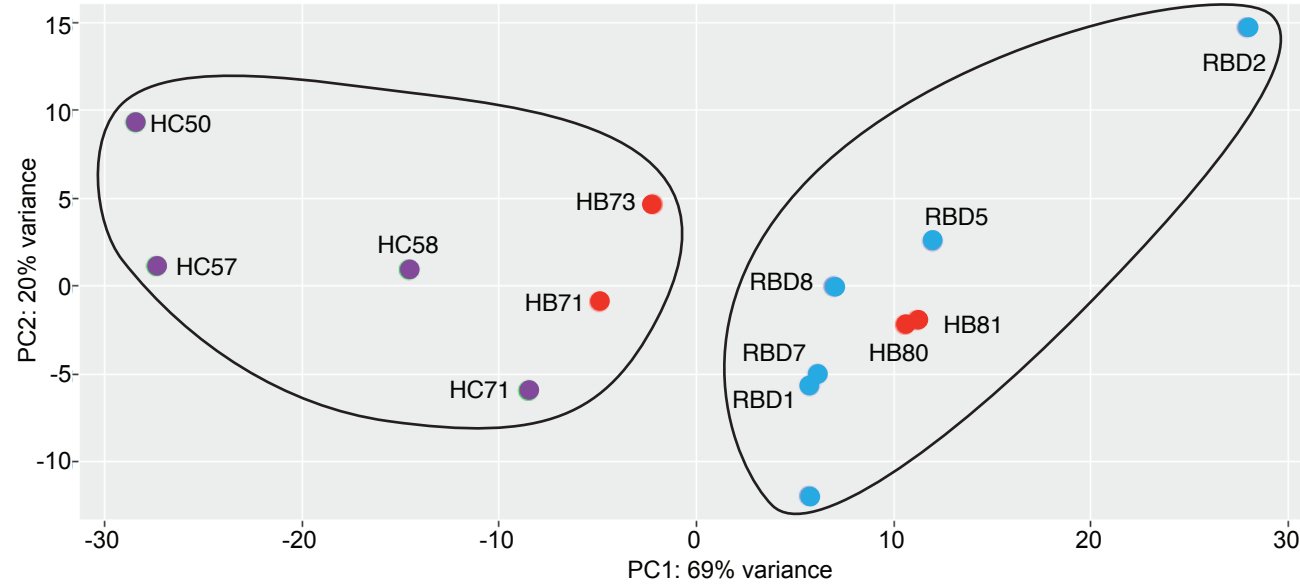

B. Cluster analysis

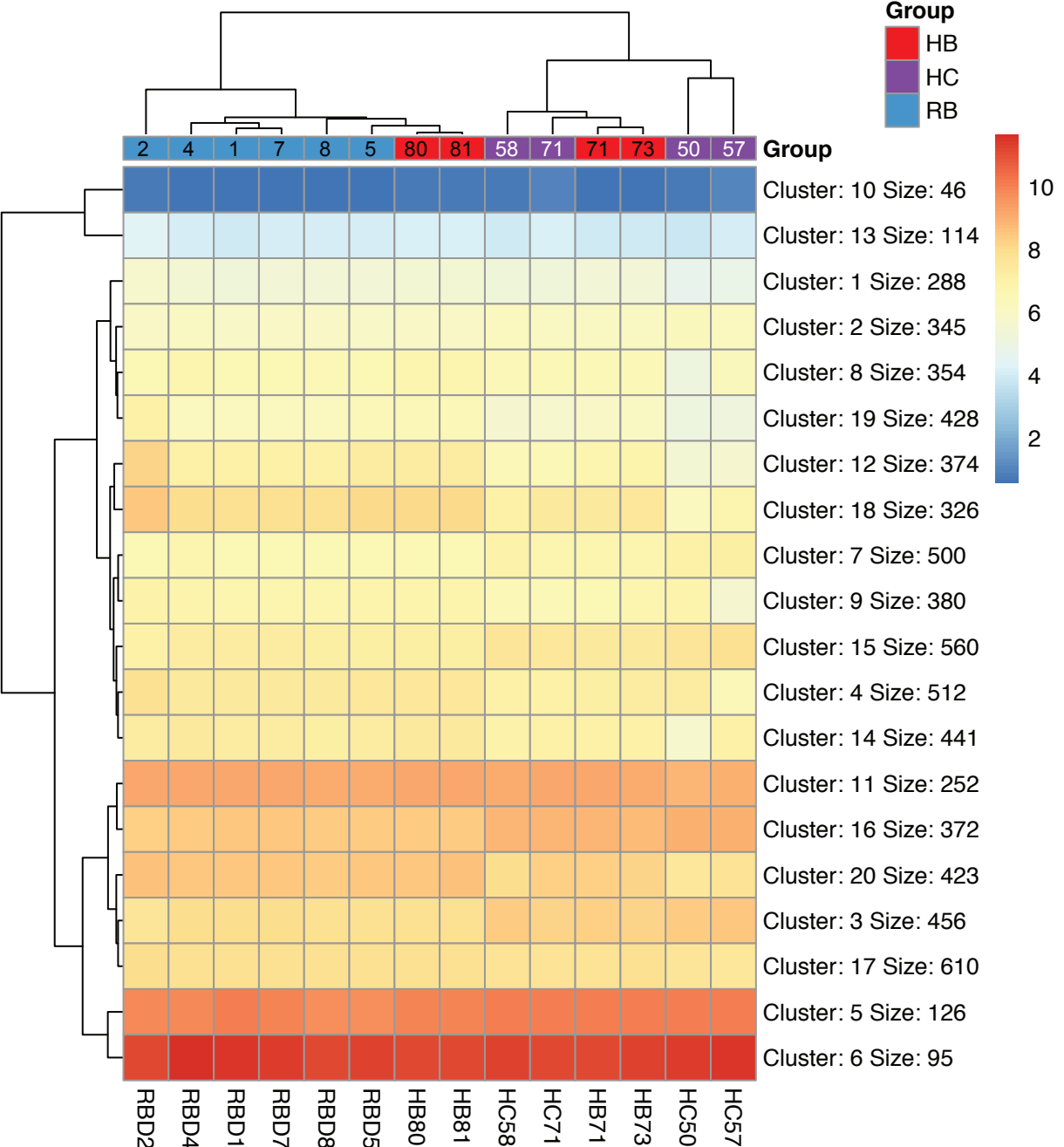

Supplement: S5 Fig — A. Principal component analysis. B. Clustering of genes according to differences in expression. The genes in each cluster are in S3 Table. See the trypclusterviewer (S1 Folder) for details. (PDF) [file pntd.0006280.s009.pdf]

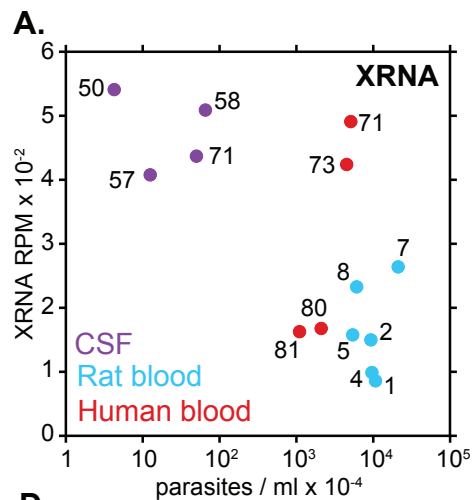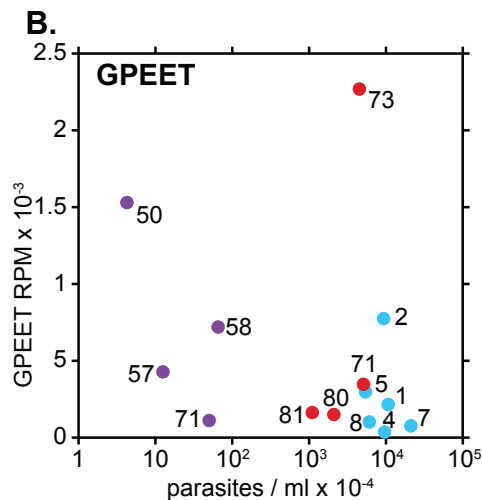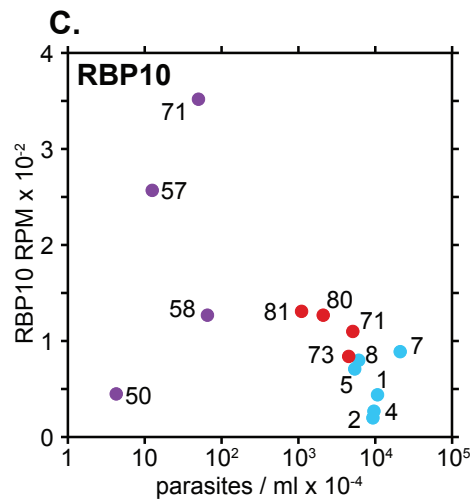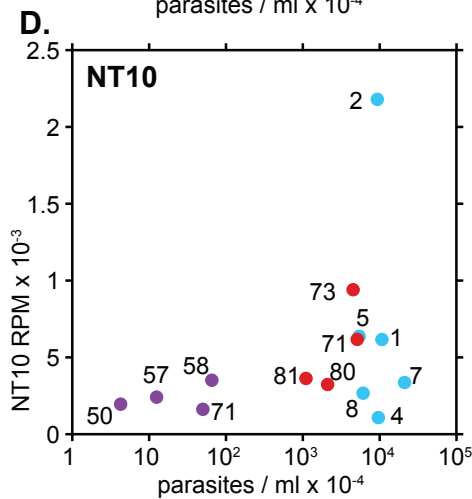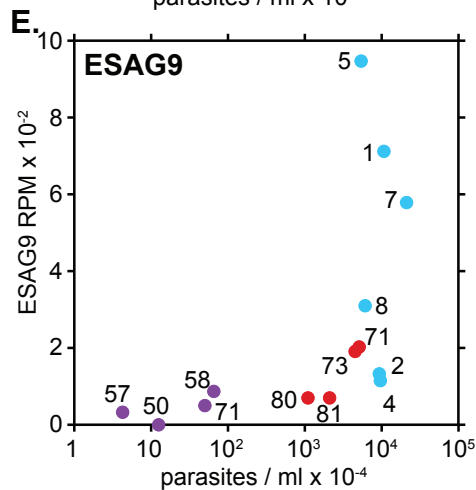

Supplement: S6 Fig — Extra panels like Fig 6, with different genes. (PDF) [file pntd.0006280.s010.pdf]
