# Supplementary material for: Transcriptomes of Trypanosoma brucei rhodesiense from sleeping sickness patients, rodents and culture: Effects of strain, growth conditions and RNA preparation methods
Source: PLoS Negl Trop Dis. 2018 Feb 23;12(2):e0006280. doi: 10.1371/journal.pntd.0006280 (PMC5842037; doi:10.1371/journal.pntd.0006280)
Supplement: S1 Folder — (ZIP) [file pntd.0006280.s011.zip › S1_folder/How to set up a new comparison.rtf]

Before you start:Update your UniqueList if necessary, as tab-separated text.Put in the relative expression values in a file called rldDF, again as as tab-separated text. This file is obtained from the previous DeSeq2 analysis. If you use DESeqUI, you can obtain the file from the “Content” tab.In the cluster programme code itself you have to put in the column headings on line 79. This will be the same list as you used for the DeSeq2 analysis to get the PCA plots and two-condition comparisons. All replicates for a given condition should have the same name, it is a comma-separated list.After that, click “run document”.If your samples fall into groups you may decide that you want to rearrange the columns so that similar samples are together, so it looks prettier (and clearer). In this case you will change your file “rldDF” and correspondingly change the column heading order on line 79 of the code.
